# Once-daily OROS® hydromorphone for the management of chronic nonmalignant pain: a dose-conversion and titration study

**DOI:** 10.1111/j.1742-1241.2007.01500.x

**Published:** 2007-10

**Authors:** M Wallace, R L Rauck, D Moulin, J Thipphawong, S Khanna, I C Tudor

**Affiliations:** 1 San Diego Medical Center, University of California La Jolla, CA, USA; 2 Wake Forest University School of Medicine, The Center for Clinical Research, Carolinas Pain Institute Winston-Salem, NC, USA; 3 London Regional Cancer Program London, ON, Canada; 4 ALZA Corporation Mountain View, CA, USA

## Abstract

**Summary:**

**Background:**

The use of opioid analgesics for patients with chronic nonmalignant pain is becoming more widely accepted, and long-acting formulations are an important treatment option.

**Aim:**

To assess conversion to extended-release OROS® hydromorphone from previous stable opioid agonist therapy in patients with chronic nonmalignant pain of moderate-to-severe intensity.

**Methods:**

In this open-label multicentre trial, patients were stabilised on their previous opioid therapy before being switched to OROS® hydromorphone at a ratio of 5 : 1 (morphine sulphate equivalent to hydromorphone hydrochloride). The OROS® hydromorphone dose was titrated over 3–16 days to achieve effective analgesia, and maintenance treatment continued for 14 days.

**Results:**

Study medication was received by 336 patients; 66% completed all study phases. Stabilisation of OROS® hydromorphone was achieved by 94.6% of patients, the majority in two or fewer titration steps (mean time, 4.2 days). Mean pain intensity scores, as determined by the Brief Pain Inventory, decreased during OROS® hydromorphone treatment (p ≤ 0.001). The percentage of patients rating their pain relief as ‘good’ or ‘complete’ increased, and the use of rescue analgesics for breakthrough pain decreased. The interference of pain with everyday activities (e.g. walking or work), and the effects on mood and enjoyment of life, also improved during the study (all p < 0.001). OROS® hydromorphone was well tolerated, and adverse events were those expected for opioid agonist therapy.

**Conclusion:**

Patients with chronic nonmalignant pain who had been receiving opioid therapy easily underwent conversion to OROS® hydromorphone, with no loss of efficacy or increase in adverse events.

**What's known**

In contrast to immediate-release hydromorphone, OROS® hydromorphone produces relatively constant steady-state concentrations for 24 h. Conversion from previous opioid therapy can be easily achieved without loss of pain control or increase in adverse events using a morphine equivalents : OROS® hydromorphone conversion ratio of 5 : 1 (based on preliminary results from a pooled analysis of two open-label, repeated-dose studies; one in subjects with malignant pain and one in subjects with nonmalignant pain).

**What's new**

The current report presents final results of the individual study conducted in patients with nonmalignant pain. It demonstrates that conversion can be achieved without loss of pain control or increase in adverse events, and provides additional evidence of the safety and efficacy for the proposed 5 : 1 morphine equivalents : OROS® hydromorphone conversion ratio.

## Introduction

Although the treatment of chronic pain with opioid agonists in cancer patients is widely accepted, the use of opioid analgesics for patients with nonmalignant pain has become widespread only in recent years, and remains controversial (1,2). However, numerous studies have shown that opioid agonists can safely and effectively control chronic pain of moderate-to-severe intensity (1,2).

Hydromorphone is a hydrogenated semi-synthetic ketone of morphine that has been used for many years to treat moderate-to-severe cancer pain, for which it is regarded as an effective alternative to morphine (3,4). Hydromorphone is available as an oral formulation, but the short elimination half-life of this drug (5) necessitates repeated dosing every 4–6 h for effective around-the-clock pain control. This need for frequent dosing could lead to reductions in compliance, which in turn could negatively impact treatment outcomes and quality of life (6,7). The introduction of long-acting opioid agonist formulations has provided an important treatment option for patients with chronic pain, and studies have shown that long-acting opioids can improve pain management and reduce opioid-related side effects in comparison to immediate-release (IR) formulations (7).

An OROS® formulation of hydromorphone was developed recently. It utilises OROS® Push-Pull™ osmotic pump technology (ALZA Corporation, Mountain View, CA) to release hydromorphone at a controlled rate for up to 24 h. Clinical pharmacokinetic analysis has shown that, in contrast to IR hydromorphone, OROS® hydromorphone produces relatively constant steady-state concentrations for 24 h with much less peak-to-trough variation (8).

The conversion of patients to OROS® hydromorphone from previous opioid agonist treatment was evaluated in an open-label, repeated-dose study. Preliminary results were presented as part of a pooled analysis, with a second study of identical design performed in patients with chronic cancer pain (9). The current report presents final results of the individual study in patients with nonmalignant pain.

## Methods

### Study design

The study was an open-label, repeated-dose, single-treatment, multicentre trial conducted at 35 sites throughout the USA and Canada. Its protocol was approved by an Institutional Review Board at each site, and all patients provided written informed consent.

Before the administration of study medication, patients were stabilised on their previous opioid therapy for at least 3 days. Stabilisation was defined as ≥ 3 consecutive days when the total daily baseline opioid dose remained unchanged, with ≤ 3 doses of rescue medication required for breakthrough pain. Patients were permitted to receive combinations of opioid drugs during this phase, and nonopioid and adjuvant analgesics also were allowed.

When stabilised, patients were switched to OROS® hydromorphone hydrochloride using a 5 : 1 conversion ratio of morphine sulphate equivalent to hydromorphone hydrochloride (4,10,11,12), with no washout or overlap of previous opioid therapy and study medication. Patients stabilised on transdermal fentanyl were converted on the basis of 8 mg/day hydromorphone for each 25 μg/h of fentanyl, which conservatively approximates the 5 : 1 conversion ratio (13). For all patients, the minimum starting dose of OROS® hydromorphone was 8 mg/day.

After the switch, OROS® hydromorphone was titrated over a period of 3–16 days. Each dose of OROS® hydromorphone was given for at least 2 days to ensure that steady-state blood levels of hydromorphone were achieved (14). If more than two doses (or 7 mg) of rescue medication (IR hydromorphone) were required in a 24-h period, the dose of OROS® hydromorphone was increased by 25 to 100%. Patients who did not achieve stable OROS® hydromorphone dosing after 21 days were discontinued from the study. After stabilisation on OROS® hydromorphone, patients entered a 14-day maintenance phase. Those who withdrew from the study had their OROS® hydromorphone dose tapered over several days (50% reduction every 2 days) until discontinuation.

Patients were treated on an outpatient basis, with five study visits over the treatment period. Stabilisation of prior opioid therapy began at visit 1 (baseline evaluation). OROS® hydromorphone titration started at visit 2, and OROS® hydromorphone maintenance therapy began at visit 3. Visits 4 and 5 occurred at the midpoint and end of the maintenance phase, respectively.

During the study, IR hydromorphone could be prescribed as rescue medication for breakthrough pain, with the recommended dose generally ranging from 10% to 15% of the basic daily OROS® hydromorphone dose. No other opioid medication was permitted after conversion; however, patients were allowed to use nonopioid and adjuvant analgesics.

### Patients

Study patients were adults (≥ 18 years of age) with chronic nonmalignant pain and stable analgesic requirements (daily opioid requirement of ≥ 45 mg morphine equivalents). Exclusion criteria included hypersensitivity to hydromorphone or other opioid agonists; gastrointestinal disorders that could affect the intake, absorption or transit of study medication; significant disorders of the central nervous system; respiratory compromise; risk of serious decrease in blood pressure with an opioid analgesic; significant organ or metabolic dysfunction; a history of drug or alcohol abuse; requirement for radiation treatment; pregnancy or lactation; and use of any investigational drug within 30 days of study initiation.

### Assessments

The efficacy of OROS® hydromorphone was assessed using the Short Form of the Brief Pain Inventory (BPI) (15). Pain intensity (worst, least and average) over the previous 24 h was rated by patients on a scale of 0 (no pain) to 10 (pain as bad as you can imagine). Pain relief was rated on a scale of 0% (no relief) to 100% (complete relief). The degree to which pain interfered with general activity, mood, walking ability, normal work, relationships with others, sleep and enjoyment of life was rated on a scale of 0 (no interference) to 10 (complete interference). In addition, both patients and physicians rated the general effectiveness of study medication on a five-point scale (1, poor; 2, fair; 3, good; 4, very good; 5, excellent). Safety monitoring was performed by recording adverse events and study discontinuations throughout the trial. Patients also underwent physical examination at baseline and end-point.

### Statistical analysis

Changes in mean BPI pain intensity, pain relief and pain interference ratings from visit 2 to end-point (or the last observation carried forward from visit 3 or 4 for patients who withdrew) were assessed using the Wilcoxon signed rank test. To detect the smallest differences in BPI ratings, a type I (*α*) error of 0.05 was assumed. Statistical significance was set at p ≤ 0.05. No adjustment for multiple testing was applied.

## Results

### Patients

**Figure 1:**
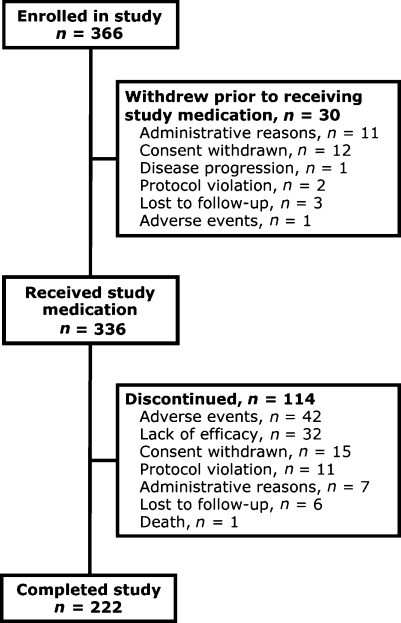
Patient disposition

Overall, 366 patients with chronic nonmalignant pain were enrolled; 336 of these received study medication (30 withdrew during the prior opioid stabilisation phase). The study was completed by 222 patients (66%); 94 (28%) discontinued during the titration phase and 20 (8%) during the maintenance phase. Patient disposition is illustrated in Figure 1, and baseline characteristics of treated patients are shown in Table 1.

**Table 1:** Demographic and baseline characteristics of treated patients

	OROS® hydromorphone (*n*=336)
Mean ± SD age, years	48.2 ± 11.7
**Sex, *n* (%)**	
Male	151 (45)
Female	185 (55)
Mean ± SD height (cm)	170.6 ± 11.0
Mean ± SD weight (kg)	81.4 ± 22.0
**Type of pain, *n* (%)**	
Sympathetic	16 (5)
Musculoskeletal	174 (52)
Neuropathic	134 (40)
Other	12 (4)
**Location of pain, *n* (%)**	
Back	195 (58)
Limbs	179 (53)
Face/head/neck	64 (19)
Torso	57 (17)
**Previous opioid medication, *n* (%)**	
Codeine	10 (3)
Fentanyl	22 (7)
Hydrocodone	46 (14)
Hydromorphone	23 (7)
Meperidine	1 (0.3)
Methadone	21 (6)
Morphine	72 (22)
Oxycodone	133 (40)
Propoxyphene	6 (2)
Mean daily opioid requirement (mg)*	154.5

*Morphine equivalent at end of stabilisation phase.

### OROS® hydromorphone dosing

The overall mean ± SD duration of OROS® hydromorphone treatment was 24.2 ± 12.2 days, with 306 patients (91%) receiving study medication for more than 7 days and 143 patients (43%) receiving it for more than 25 days.

**Figure 2:**
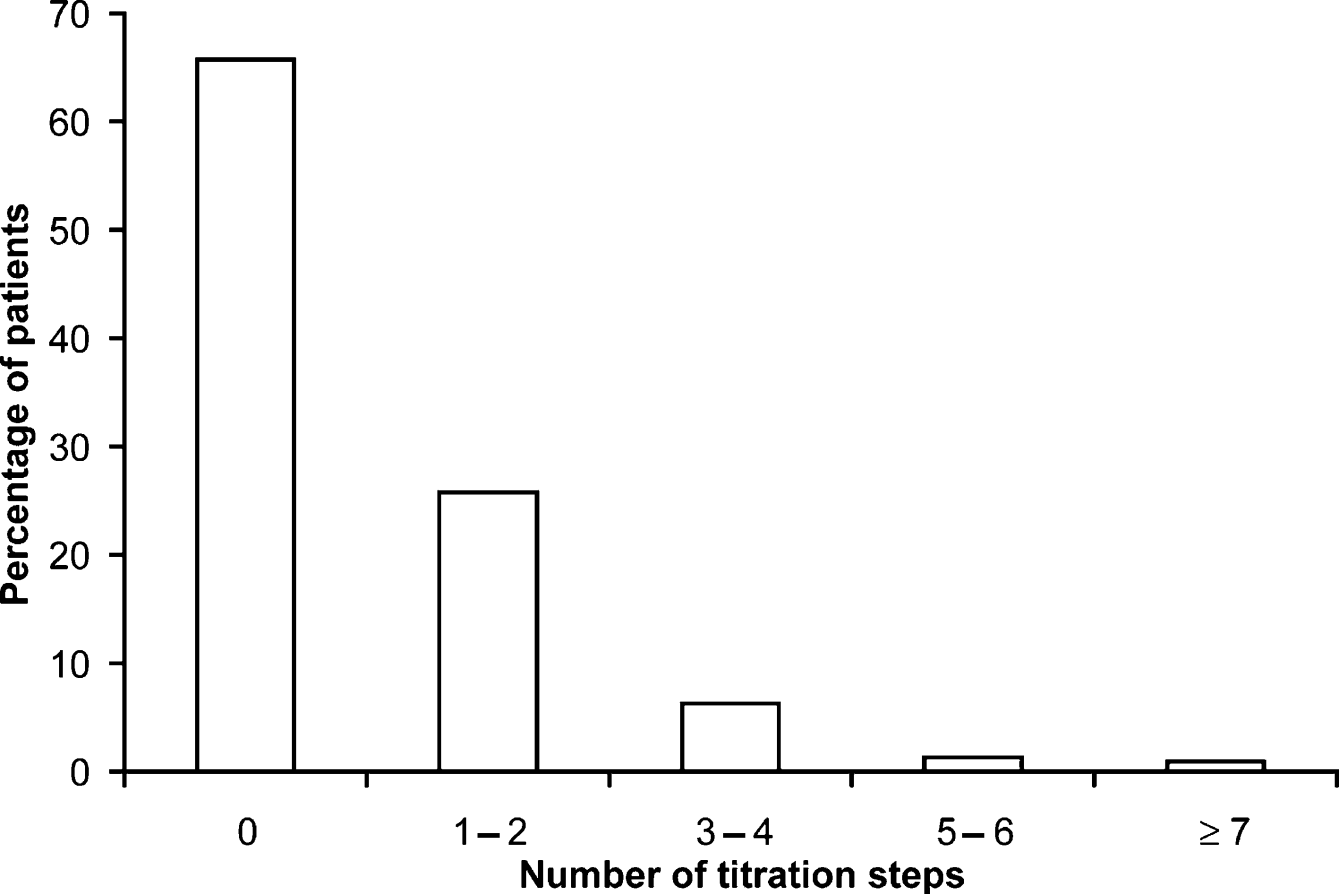
Percentage of patients requiring titration steps to achieve stabilisation of OROS® hydromorphone dose (*n* = 318)

Three hundred and eighteen of the 336 treated patients (94.6%) achieved a stable dose of OROS® hydromorphone during the titration phase. Overall, the mean time to dose stabilisation was 4.2 ± 2.12 days. Stabilisation was achieved in two or fewer titration steps by 87% of patients [no titration, *n* = 209 (66%); one or two titration steps, *n* = 82 (26%); Figure 2]. The mean ± SD dose of prior opioid therapy at the end of the prior opioid stabilisation phase was 154.5 ± 172.6 mg morphine equivalents, and the mean starting daily dose of OROS® hydromorphone was 30.1 ± 37.9 mg, resulting in a mean conversion ratio of 5.13 : 1. The mean daily dose of OROS® hydromorphone increased to 56.6 ± 63.3 mg at the end of titration and to 70.1 ± 146.1 mg at the end of the maintenance phase.

At the start of the OROS® hydromorphone titration period, mean rescue medication requirements were 4.8 ± 4.4 doses per day and 15.7 ± 20.5 mg per day. The frequency of rescue medication use, but not mean dose, declined during OROS® hydromorphone therapy (Table 2).

**Table 2:** Rescue medication (IR hydromorphone) used by patients receiving daily OROS® hydromorphone

	No. daily doses	Daily dose (mg)
**Start of titration**		
Mean ± SD	4.8 ± 4.36	15.7 ± 20.47
**End of titration**		
Mean ± SD	4.0 ± 6.04	14.3 ± 20.47
Mean change	−0.9	−2.1
**End of maintenance**		
Mean ± SD	4.1 ± 10.38	15.8 ± 42.54
Mean change	−0.5	0.8

### Analgesic efficacy

**Figure 3:**
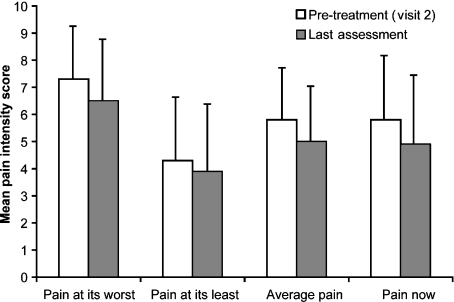
Comparison of BPI pain intensity ratings: end of previous opioid stabilisation phase vs. end of treatment. Scale: 0, no pain; 10, pain as bad as you can imagine (p ≤ 0.001 for all scores; no adjustment for multiple testing)

Mean BPI pain intensity ratings for pain at its worst, pain at its least, average pain and pain right now all decreased with OROS® hydromorphone treatment (from the end of previous opioid stabilisation to the end of OROS® hydromorphone treatment; all p ≤ 0.001) (Figure 3). Patients’ assessment of pain relief (BPI) also improved during OROS® hydromorphone treatment, from 52.7 ± 22.5% at the end of previous opioid stabilisation to 60.1 ± 23.9% at last postbaseline assessment.

**Figure 4:**
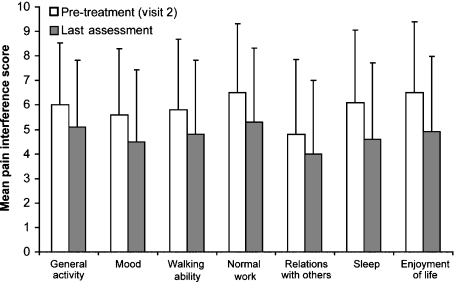
Comparison of BPI pain interference ratings: end of previous opioid stabilisation phase vs. end of treatment. Scale: 0, no interference; 10, complete interference (p < 0.001 for all scores; no adjustment for multiple testing)

In addition to the improvement in pain intensity, patients reported a decrease in the degree to which pain interfered with their lives, as assessed by BPI pain interference scores (all p < 0.001) (Figure 4). Investigators rated the overall effectiveness of medication as very good or excellent for 9% of patients at baseline (visit 2) and for 27% at the last assessment. Similarly, more patients rated the overall effectiveness of medication as very good or excellent at the last assessment (26%) than at the beginning of OROS® hydromorphone titration (8%).

### Safety and tolerability

During the study, adverse events were experienced by 79% of patients. The most common events were gastrointestinal symptoms (such as nausea, constipation and vomiting) as well as headache, dizziness and somnolence (Table 3). The majority of these events were mild or moderate in severity. Serious adverse events were reported by 13 patients (4%), three of whom experienced episodes of overdose related to patient noncompliance with therapy. Two of these three patients were withdrawn from the study, and all three recovered with no sequelae. No other serious adverse events were considered probably or definitely related to study treatment. One death occurred during the study, which resulted from a perforated ulcer in the caecum of a patient with a history of morbid obesity. This event was not considered related to the study treatment. No clinically significant changes in vital signs occurred during the trial.

**Table 3:** Adverse events occurring in ≥ 5% of treated patients (*n* = 336)

Event	No. patients (%)
Any adverse event	264 (79)
Nausea	79 (24)
Headache	61 (18)
Constipation	60 (18)
Dizziness	54 (16)
Vomiting	51 (15)
Somnolence	49 (15)
Pruritus	28 (8)
Sweating	22 (6)
Insomnia	20 (6)
Dry mouth	20 (6)
Diarrhoea	19 (6)
Fatigue	18 (5)
Peripheral oedema	17 (5)

## Discussion

Results of the previous interim pooled analysis showed that patients with chronic malignant or nonmalignant pain could be switched easily from previous opioid therapy to OROS® hydromorphone (9). This finding was confirmed by the final results of the present study in patients with chronic nonmalignant pain. The majority of treated patients (∼95%) achieved a stable dose of OROS® hydromorphone, most of them (87%) in two or fewer titration steps (4 days).

The efficacy results of the present study indicate that conversion from previous opioid therapy to OROS® hydromorphone can be achieved with no loss of pain control. Over the course of OROS® hydromorphone treatment, patients’ BPI pain intensity ratings (worst, least and average pain, and pain right now) improved significantly (p ≤ 0.001) in comparison to the ratings recorded for their previous opioid therapy. The improvement in pain interference was similar. At the end of the maintenance period, there were statistically significant (p < 0.001) decreases in the degree to which patients’ pain interfered with general activity, mood, ability to walk, normal work, relationships with other people, sleep and general enjoyment of life. Such improvement can be expected to positively impact quality of life, as has been demonstrated for extended-release formulations of opioid agonists (7). A German study conducted with a sustained-release formulation of hydromorphone (Palladone) in 487 patients showed improvement in quality of life domains, such as sleep, vitality, mood, social contacts, activity, resilience and walking ability (16), consistent with results of the present study.

As has been demonstrated previously with extended-release morphine and oxycodone (17,18), conversion to OROS® hydromorphone was achieved without the need for an intermediate IR opioid phase. The conversion ratio used in the study protocol is based on the commonly used 5 : 1 ratio of morphine equivalents to hydromorphone (4,10,11,12). However, some investigators, including the American Pain Society, recommend a reduction in equivalent dose when introducing a new long-acting opioid into treatment (19). The results of the present study demonstrate that most patients can successfully undergo direct conversion from their previous opioid agonist therapy to OROS® hydromorphone using the 5 : 1 ratio, with no loss of efficacy or increase in side effects. Moreover, in the majority of patients, conversion at this level requires little or no titration to achieve stabilisation of the OROS® hydromorphone dose. Interestingly, even fewer patients required dose titration in the study of identical design in patients with chronic cancer pain (104 manuscript in preparation). In that study, 77% of patients achieved a stable OROS® hydromorphone dose with no titration, and another 20% required only one to two titration steps. In an analysis of two studies in which an 8 : 1 ratio was used for conversion, 70% of patients achieved a stable dose of hydromorphone (20).

OROS® hydromorphone was well tolerated throughout the study. The most common adverse events affected the gastrointestinal and central nervous systems, and were consistent with those expected to occur with opioid agonist therapy for chronic pain.

Limitations of the present study include the open-label design and the lack of a control group. However, this study does show that patients can be switched easily from stable opioid agonist therapy to OROS® hydromorphone, and that titration of OROS® hydromorphone will enable achievement of a stable dose that maintains effective pain control. The 5 : 1 conversion ratio (morphine equivalents to hydromorphone) also was well tolerated, with adverse events typical of those expected for OROS® opioid agonist therapy. Based on these promising results, the role of OROS® hydromorphone in the treatment of chronic nonmalignant pain should be further assessed by prospective, randomised and controlled trials.
